# The role of statins in amyotrophic lateral sclerosis: protective or not?

**DOI:** 10.3389/fnins.2024.1422912

**Published:** 2024-06-05

**Authors:** Hayder M. Al-kuraishy, Majid S. Jabir, Ghassan M. Sulaiman, Hamdoon A. Mohammed, Ali I. Al-Gareeb, Ali K. Albuhadily, Sabrean F. Jawad, Ayman A. Swelum, Mosleh M. Abomughaid

**Affiliations:** ^1^Department of Clinical Pharmacology and Medicine, College of Medicine, Mustansiriyah University, Baghdad, Iraq; ^2^Department of Applied Sciences, University of Technology, Baghdad, Iraq; ^3^Department of Medicinal Chemistry and Pharmacognosy, College of Pharmacy, Qassim University, Qassim, Saudi Arabia; ^4^Department of Pharmacognosy and Medicinal Plants, Faculty of Pharmacy, Al-Azhar University, Cairo, Egypt; ^5^Department of Clinical Pharmacology and Medicine, College of Medicine, Jabir Ibn Hayyan Medical University, Kufa, Iraq; ^6^Department of Pharmacy, Al-Mustaqbal University College, Hillah, Iraq; ^7^Department of Animal Production, King Saud University, Riyadh, Saudi Arabia; ^8^Department of Medical Laboratory Sciences, College of Applied Medical Sciences, University of Bisha, Bisha, Saudi Arabia

**Keywords:** Amyotrophic lateral sclerosis, neurodegenerative diseases, pathogenesis, statins, aging

## Abstract

Amyotrophic lateral sclerosis (ALS) is a progressive neurodegenerative disease of motor neurons characterized by muscle weakness, muscle twitching, and muscle wasting. ALS is regarded as the third-most frequent neurodegenerative disease, subsequent to Alzheimer's disease (AD) and Parkinson's disease (PD). The World Health Organization (WHO) in 2007 declared that prolonged use of statins may induce development of ALS-like syndrome and may increase ALS risk. Subsequently, different studies have implicated statins in the pathogenesis of ALS. In contrast, results from preclinical and clinical studies highlighted the protective role of statins against ALS neuropathology. Recently, meta-analyses and systematic reviews illustrated no association between long-term use of statins and ALS risk. These findings highlighted controversial points regarding the effects of statins on ALS pathogenesis and risk. The neuroprotective effects of statins against the development and progression of ALS may be mediated by regulating dyslipidemia and inflammatory changes. However, the mechanism for induction of ALS neuropathology by statins may be related to the dysregulation of liver X receptor signaling (LXR) signaling in the motor neurons and reduction of cholesterol, which has a neuroprotective effect against ALS neuropathology. Nevertheless, the exact role of statins on the pathogenesis of ALS was not fully elucidated. Therefore, this narrative review aims to discuss the role of statins in ALS neuropathology.

## 1 Introduction

Amyotrophic lateral sclerosis (ALS), also known as Lou Gehrig's disease, is a progressive neurodegenerative disease among motor neuron diseases (Verber et al., 2019; Feldman et al., 2022). Other motor neuron diseases include primary lateral sclerosis, progressive muscle atrophy, progressive bulbar palsy, monometic amyotrophy, and pseudobulbar palsy (Verber et al., 2019). ALS was first described by Charles Bell in 1824; subsequently, in 1869, Jean-Martin Charcot explained the association between neurological disorders and the presenting feature of ALS; and the term ALS was first used by Jean-Martin Charcot in 1874 (Cho and Shukla, 2020).

ALS is the most common type of motor neuron diseases and is the third-most frequent neurodegenerative disease subsequent to Alzheimer's disease (AD) and Parkinson's disease (PD) (Bennett et al., 2019; Masrori and Van Damme, 2020). The incidence of ALS is more common in men of White race and those with age >60 (Mehta et al., 2022). It has been reported that ALS prevalence is 50–100 times higher in the Western Pacific regions than other regions in the world (Mehta et al., 2022). ALS can occur at any age, but its peak incidence is between 50 and 75 years and sharply decreases after 80 years (Vinceti et al., 2019). Moreover, sporadic ALS is more common in people aged 58–63 years. ALS cases are expected to reach 300,000 in 2040 worldwide (Vinceti et al., 2019; Mehta et al., 2022).

ALS is characterized by muscle weakness, muscle twitching, and muscle wasting. In limb-onset ALS, the muscle weakness starts in the arms or legs in 50% of patients. The weakness most commonly starts in the limb muscles, more often in distal muscles than in proximal muscles (Bennett et al., 2019; Masrori and Van Damme, 2020). In bulbar-onset ALS, there are difficulties in swallowing and speaking, which are present in 25%−30% of ALS patients (Bennett et al., 2019). Approximately 50% of patients will suffer from extra-motor manifestations to some degree in addition to their motor problems (Masrori and Van Damme, 2020). It has been observed that 15% of ALS patients develop frontotemporal dementia and cognitive impairments (Masrori and Van Damme, 2020). According to the etiopathological factors, there are two types of ALS: 95% is idiopathic (sporadic) ALS and 5% is familial ALS (Delpont et al., 2019; Masrori and Van Damme, 2020).

Classical ALS involves both upper and lower motor neurons, leading to spasticity and muscle weakness, respectively. Classical ALS contributes to 70% of all ALS cases classified as limb-onset ALS and bulbar-onset ALS. Some ALS variants present limited symptoms due to localized neuronal injury that progresses slower than classical ALS, such as flail arm syndrome, caused by damage to motor neurons in arm muscles (Masrori and Van Damme, 2020). In addition, isolated bulbar palsy is caused by damage to motor neurons in the bulbar region, leading to dysarthria and dysphagia (Delpont et al., 2019; Masrori and Van Damme, 2020; Goutman et al., 2022). The diagnosis of ALS is based on clinical manifestations and neuroimaging findings (Goutman et al., 2022).

ALS is an incurable disease, and the treatment is mainly symptomatic. Median survival time is 2–4 years from onset; only 5%−10% of patients survive beyond 10 years (Chiò et al., 2013). The ALS mortality rate was 1.70 per 100,000 in the United States. Similarly, other studies have also found lower ALS mortality rates among Hispanics. Lower incidence and mortality among non-Whites and Hispanics may reflect less detection among non-Whites or a genetic factor predisposing Whites to ALS (Goutman et al., 2022). However, slowing the progression of the disease is the main goal in ALS management by using riluzole and sodium phenylbutyrate, which extend the life only by several months (Chiò et al., 2013; Andrews et al., 2020).

The causes of ALS are not fully known; however, environmental and genetic factors contribute equally to the pathogenesis of ALS (Feldman et al., 2022; Hartmann et al., 2022). Genetic alterations, together with environmental factors, promote the development of ALS (Motataianu et al., 2022). ALS below the age of 70 is more common in men by 20%, but sex differences are not observed above the age of 70 years (Borghero et al., 2023). ALS neuropathology is characterized by neuronal lesions located in the brain motor cortex for the upper motor neurons, the brain stem, and spinal cord for the lower motor neurons (Del Tredici and Braak, 2022). The pathognomonic pathological feature of ALS is the presence of specific inclusion bodies called Bunina bodies in the cytoplasm of motor neurons (Bede et al., 2021).

The constituents of Bunina bodies are TAR DNA-binding protein 43 (TDP-43), which is present in 97% of ALS patients (Mori et al., 2019). However, in ALS, superoxide dismutase 1 (SOD1) is the main constituent of Bunina bodies, which is due to a gene mutation (Wright et al., 2019). These inclusion bodies have the ability to transport from cell to cell in a prion disease-like manner (Mori et al., 2019; Wright et al., 2019). Mutant SOD1 proteins have the ability to accumulate intracellularly and inhibit protein degradation (Berdyński et al., 2022). Inclusion bodies in ALS induce glutamate neurotransmission, leading to excessive intracellular calcium accumulation and excitotoxicity (Diana and Bongioanni, 2022). It has been shown that motor neurons are highly sensitive to the effect of excitotoxicity due to their low calcium buffering capacity (Tedeschi et al., 2021).

In ALS, excitatory amino acid transporter 2 (EAAT2), which is responsible for the transport of synaptic glutamate, is significantly downregulated (Fontana et al., 2023). Therefore, dysregulation of glutamate neurotransmission is regarded as the main pathophysiological mechanism intricate in ALS neuropathology. Hence, riluzole, which inhibits presynaptic glutamate release, can attenuate the pathogenesis of ALS (Chen et al., 2020). Interestingly, environmental factors such as toxins, viruses, and metals, together with SOD-1 mutation, trigger excitotoxicity, mitochondrial dysfunction, oxidative stress, neuroinflammation, and aggregation of abnormal proteins, leading to impairment of axonal transport and neuronal apoptosis (Mori et al., 2019; Bede et al., 2021; Del Tredici and Braak, 2022) .

According to the genetic base, TDP-43 and SOD-1 genetic mutations are the main mutations involved in the pathogenesis of ALS (Farina et al., 2021; Brenner and Freischmidt, 2022). Other mutated genes involved in ALS are listed as follows (Renton et al., 2014; Gregory et al., 2020) (Table 1).

**Table 1:** Genes involved in ALS.

**Gene**	**Location**	**Inheritance**	**Function**	**% in familial ALS**	**% in sporadic ALS**
*SQSTM1*	1q35	AD	Autophagy	1	< 1
*TARDBP*	1p36	AD	RNA metabolism	4	1
*VCP*	9p13	AD	Proteasome	1	1
*C9ORF72*	9p21	AD	DENN protein	40	1
*OPTN*	10p13	AD	Vesicle trafficking	< 1	< 1
*FUS*	16p11	AD	RNA metabolism	4	1
*PFN1*	17p13	AD	Cytoskeleton	< 1	< 1
*UBQLN2*	Xp11	XD	Proteasome	< 1	< 1

AD, autosomal-dominant; XD, X-linked dominant.

The underlying causes of cell deaths in ALS are development of mitochondrial dysfunction, impairment of nucleocytoplasmic transport, and ROS production due to progressive accumulation of mutant TDP-43 (Davis et al., 2018). TDP-43 interferes with mitochondrial respiratory complex I proteins (Wang et al., 2016), leading to mitochondrial dysfunction and loss of motor neurons in ALS. Interestingly, loss of motor neurons in ALS is mainly through induction of the apoptotic pathway (Vogt et al., 2018).

Originally, it was established that mutant TDP-43 triggers the expression of pro-apoptotic proteins in a p53-dependent manner (Vogt et al., 2018). However, neuronal death in ALS is independent of caspase-3 activation, and inhibition of caspase-3 does not affect the percentage of neuronal loss (Re et al., 2014). In addition, co-cultured astrocytes derived from ALS patients with human motor neurons led to high neuronal loss. Similarly, necroptosis is implicated in the progression of neuronal loss in ALS. The application of the necroptosis inhibitor necrostatin-1 prevents neuronal loss in ALS, which substantiates the aforementioned (Re et al., 2014). Therefore, both apoptosis and necroptosis pathways are intricately involved in neuronal loss in ALS.

Furthermore, derangement of RNA metabolism in ALS is evident in the majority of familial ALS due to alteration of RNA translocation, RNA degradation, pre-mRNA splicing, and the formation of a ribonucleoprotein complex by mutant genes (Strong, 2010). The mutant TDP-43 and SOD1 proteins can form cytosolic aggregates, which affect the stability of mRNA through an interaction with mRNA species in ALS (Strong, 2010). Therefore, ALS is regarded as a disorder of RNA metabolism, and alteration of RNA metabolism appears central to the pathogenesis of ALS. In addition, microRNA (miRNA) is a noncoding RNA that is involved in substantial control of gene expression and has a critical role in cell death. Importantly, miRNA controls apoptosis, programmed cell death, and necroptosis in ALS (Gagliardi et al., 2019). Deregulation of miRNA has been observed in the brain, spinal cord, blood, and CSF of ALS patients (Gagliardi et al., 2019). Downregulation of miRNA was observed mainly in the motor neurons (Gagliardi et al., 2019), suggesting the protective role of miRNA against ALS neuropathology.

**Figure 1:**
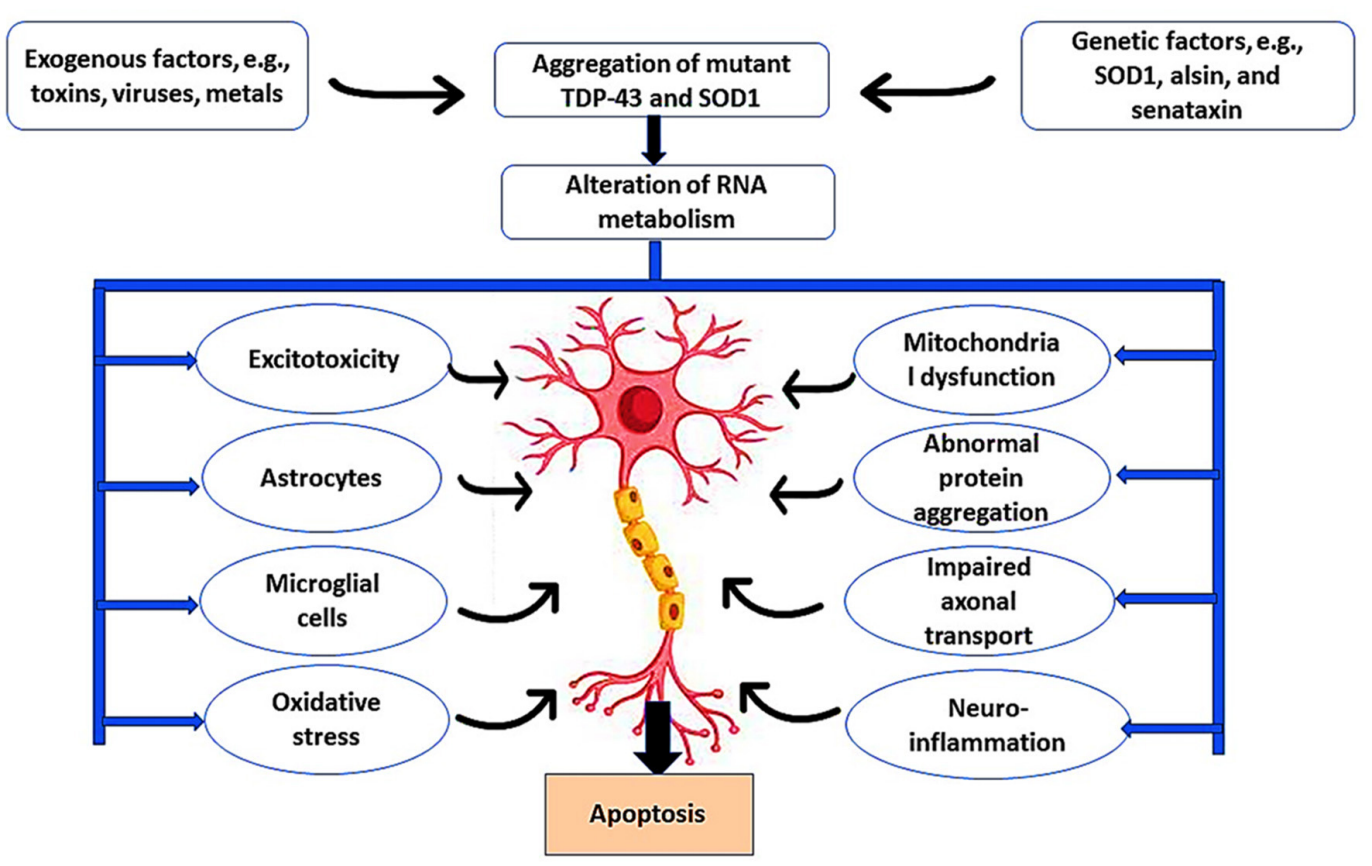
Pathophysiology of ALS. Exogenous factors such as toxins, viruses, and metals induce ALS neuropathology through the induction of microglia and astrocytes that cause excitotoxicity and oxidative stress. In addition, genetic risk factors such as mutations in the *SOD1* gene promote ALS neuropathology by inducing mitochondrial dysfunction, abnormal protein aggregation, impaired abnormal axonal transport, and development of neuroinflammation. These changes trigger the apoptosis of motor neurons.

Moreover, noncoding genomes such as long noncoding RNA (IncRNA) are highly expressed in spinal motor neurons in the early stages of the ALS pathological process (Gagliardi et al., 2018a, 2019). IncRNA acts as an epigenetic regulator of the target gene by controlling the interaction between miRNA and mRNA. It has been observed that IncRNA is upregulated in the peripheral blood mononuclear cells of sporadic ALS but deregulated in familial ALS (Gagliardi et al., 2018b). Notably, degeneration of motor neurons is caused by a combination of intrinsic neuronal defects and extrinsic factors such as astrocyte-mediated toxicity (Del Tredici and Braak, 2022). In ALS, the astrocytes are activated to release pro-inflammatory cytokines and chemokines, causing sustained neurotoxic effects and neuronal apoptosis (Bede et al., 2021), as illustrated in Figure 1.

As ALS is more common in older age groups having comorbidities such as diabetes, hypertension, and dyslipidemia, the use of lipid-lowering agents such as statins is more frequent in those patients (Hu and Ji, 2022). It has been demonstrated that statins may have beneficial or detrimental effects on the pathogenesis of ALS (Nabizadeh et al., 2022; Wang et al., 2023). Thus, this review aims to discuss the potential effects of statins on ALS neuropathology.

## 2 Method and search strategy

In this review, a systematic literature search of the Web of Science, Scopus, PubMed, Cochrane Library, ProQuest, ISI Processing, and OpenGray databases was performed to recognize the relevant literature. Additional records were obtained through a manual search. Two authors assessed the eligibility by checking the title and abstract and checking the full articles. The search was conducted by using relevant keywords [Amyotrophic lateral sclerosis AND Statins], [Amyotrophic lateral sclerosis AND Neuronal apoptosis], [Amyotrophic lateral sclerosis AND Oxidative stress], [Amyotrophic lateral sclerosis AND Neuroinflammation], [Statins AND Oxidative stress], and [Statins AND Neuroinflammation]. The quality of the studies was estimated by the authors for compatibility with the present review. The included studies are original studies, retrospective studies, and prospective studies. However, a case report study, a review study, and articles published in languages other than English were excluded from this review.

## 3 Pharmacology of statins

**Figure 2:**
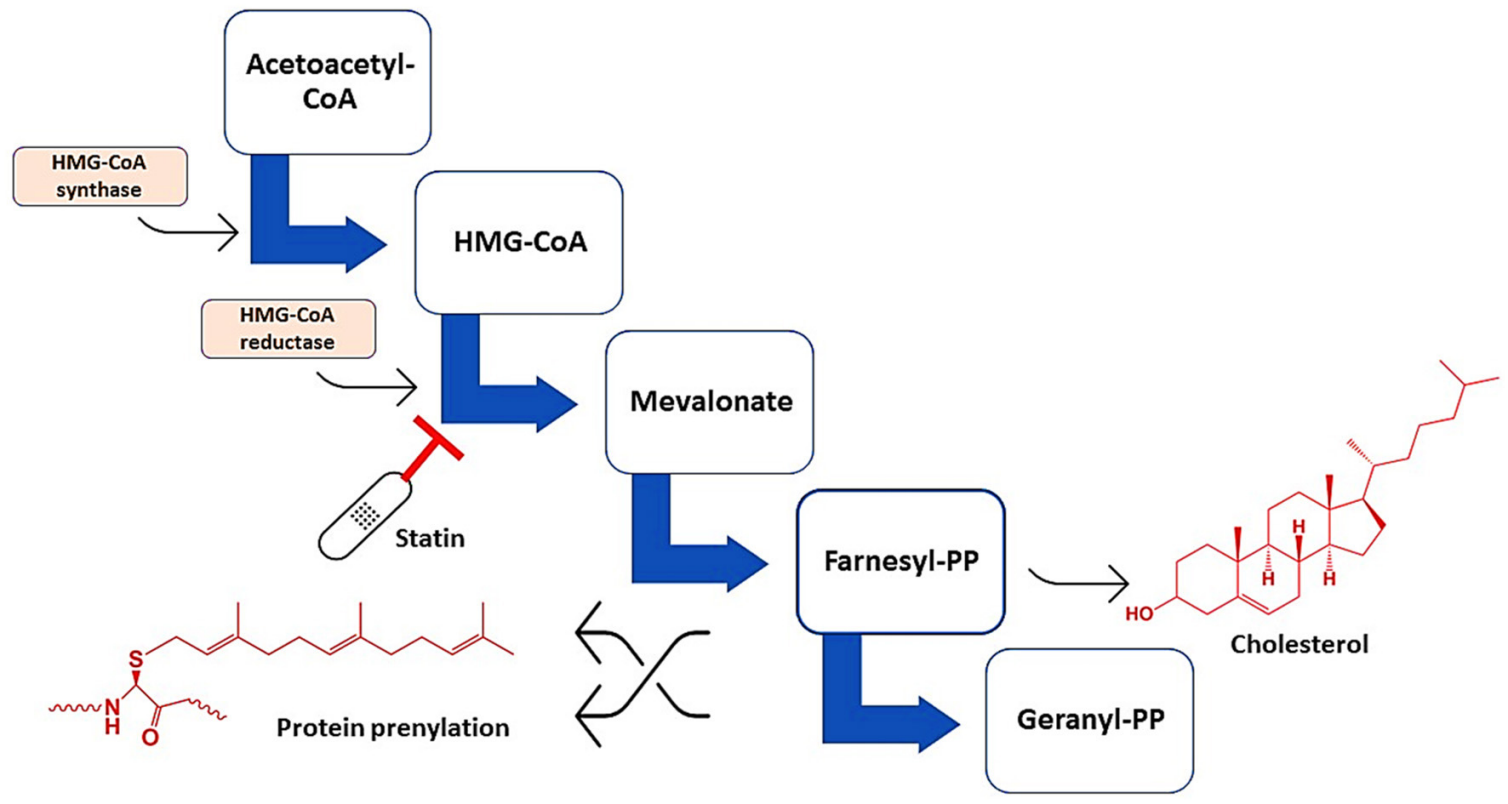
Mechanisms of action of statins. Statins inhibit HMG-CoA reductase, leading to the formation of mevalonate, farnesyl pyrophosphate, and geranyl pyrophosphate, which are essential for the synthesis of cholesterol and involved in protein prenylation.

Statins are cholesterol-lowering drugs that inhibit *de novo* hepatic cholesterol biosynthesis through suppression of a rate-limiting hydroxyl-methyl-glutaryl Coenzyme A (HMG-CoA) reductase (Al-Kuraishy et al., 2020b; Alromi et al., 2021). Figure 2 shows the mechanisms of statins.

Reduction of circulating cholesterol by statins triggers the expression of low-density lipoprotein (LDL) receptors, subsequently increasing the uptake of LDL-c (Al-Kuraishy and Al-Gareeb, 2017). In addition, statins promote the expression of high-density lipoprotein (HDL), which removes cholesterol from the tissues to the liver (Al-Kuraishy et al., 2020b). In addition, statins have potential pleiotropic effects, including antioxidant, anti-inflammatory, antiatherogenic, and antiplatelet effects (Kadhim et al., 2019; Al-Rubiay et al., 2021). Therefore, statins are used in the management of hypercholesterolemia, dyslipidemia, and as primary and secondary preventive therapy against cardiovascular diseases. According to their lipid solubility, statins are classified into lipophilic statins, such as simvastatin and atorvastatin, and hydrophilic statins, such as rosuvastatin (Al-Kuraishy et al., 2020c). Lipophilic statins can cross the blood–brain barrier (BBB) causing inhibition of brain cholesterol by suppressing the activity of brain HMG-CoA reductase (Al-Kuraishy and Al-Gareeb, 2016; Al-Kuraishy et al., 2018a).

Lipophilic statins have more central effects compared to hydrophilic statins (Simon et al., 2019; Climent et al., 2021); therefore, lipophilic statins may affect neurocognitive functions in a dual role that could be detrimental or beneficial (Jamshidnejad-Tosaramandani et al., 2022). Regarding the pharmacokinetics of statins, they are absorbed orally and may be affected by food. For example, pravastatin absorption is reduced by 35% when administrated with food, while food increases the absorption of lovastatin by 50% (Al-Kuraishy et al., 2018b). However, the absorption of rosuvastatin and pitavastatin is not affected by food (Al-Kuraishy et al., 2019). Statins have a plasma protein-binding capacity of 95%, with the exception of pravastatin, which has a plasma protein-binding capacity of 50%. Some statins can inhibit P-glycoprotein, leading to drug–drug interactions with P-glycoprotein substrates such as verapamil (Al-Kuraishy et al., 2019).

Statins are mainly metabolized by CYP450 isoenzymes, though pravastatin is metabolized by the cytosolic sulfation pathway (Filppula et al., 2021). Enzyme inducer drugs such as rifampicin increase statin metabolism, while enzyme inhibitors such as macrolides increase the risk of statin toxicity (Filppula et al., 2021). However, pitavastatin and rosuvastatin are less metabolized by CYP450 isoenzymes, so they are less subjected to drug–drug interactions (Hirota et al., 2020). In addition, P-glycoprotein is concerned with the biliary and intestinal elimination of rosuvastatin and pravastatin (Liu, 2019). Statins are mainly excreted by urine, with the exception of atorvastatin, which is eliminated through bile. The most common adverse effects linked with prolonged use of statins are gastrointestinal discomfort, myopathy, cognitive dysfunction, elevation of liver enzymes, an increased risk of diabetes, and peripheral neuropathy (Deng et al., 2021). The pharmacokinetic effects of statins are unique, intestinal SLCO2B1 protein mediates the absorption of statins, which, through the intestinal CYP3A4 enzyme, are converted to active and inactive metabolites. Statin metabolites produced by the hepatic CYP3A4 enzyme are processed and eliminated from the circulation (Hirota et al., 2020; Filppula et al., 2021).

## 4 Statins role in neurodegenerative diseases

Neurodegenerative diseases are a group of diseases caused by progressive degeneration of neurons with subsequent neuronal deaths (Hansson, 2021). Neuroinflammation, oxidative stress, and mitochondrial dysfunction are the most common mechanisms sharing an intricate link in the neurodegenerative process (Elfawy and Das, 2019). The underlying causes for development of neuroinflammation, oxidative stress, and mitochondrial dysfunction are progressive accumulation of misfolded proteins such as amyloid beta (Aβ) in Alzheimer's disease (AD) and alpha synuclein (α-Syn) in Parkinson's disease (PD) (Kabir et al., 2020). The etiopathological causes of neurodegenerative diseases are chiefly unknown; however, environmental factors in genetically susceptible subjects may be the most proposed mechanism (Al-Kuraishy et al., 2020a). AD is the most common neurodegenerative disease and represents two-thirds of dementia cases (Alromi et al., 2021; Al-Kuraishy et al., 2023b). AD is characterized by intracellular accumulation of neurofibrillary tangles (NFTs) and extracellular accumulation of non-soluble Aβ (Alsubaie et al., 2022). AD is most common in individuals aged >65 years, leading to cognitive dysfunction and progressive memory loss (Ali et al., 2024).

**Figure 3:**
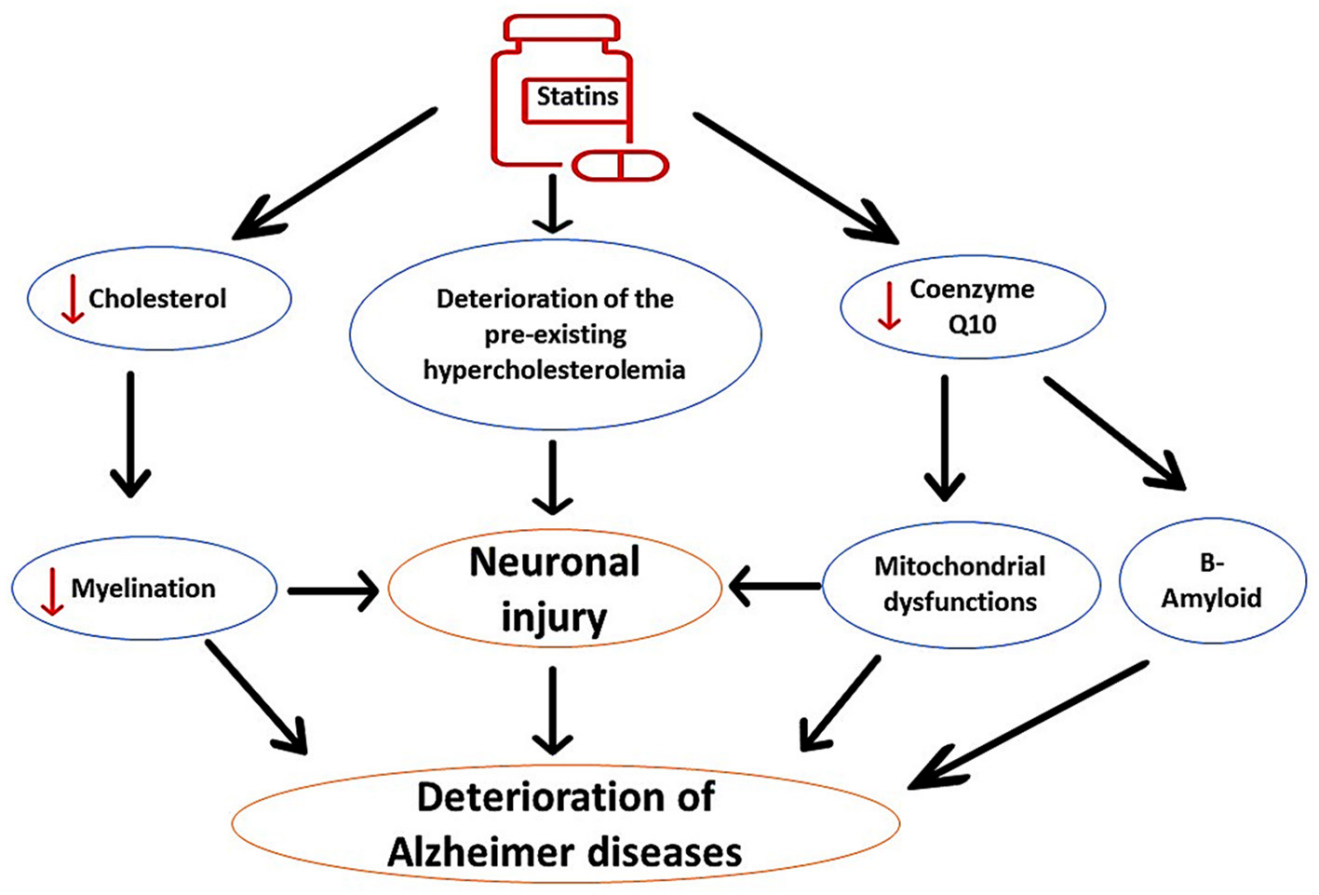
The detrimental effects of statins on AD neuropathology. Statins impair neuronal myelination through inhibition of neuronal cholesterol; therefore; statins worsen preexistent cholesterol aberrations. In addition, statins inhibit mitochondrial CoQ10, leading to oxidative stress, neuronal injury, and acceleration of Aβ aggregation. These changes induced by the use of statins exacerbate AD neuropathology.

It has been shown that prolonged use of statins, mainly in elderly patients, may adversely affect cognitive function. Statins can induce cognitive decline in healthy subjects and in AD patients (Alsubaie et al., 2022). However, observational and prospective studies did not provide a clinical clue regarding the harmful and beneficial effects of statins on AD neuropathology (Group, 2002; McGuinness et al., 2009). Interestingly, users of statins such as simvastatin and atorvastatin experience reversible cognitive impairment within 2 months (Wagstaff et al., 2003). It has been illustrated that lipophilic statins trigger the development of cognitive impairment through inhibition of brain cholesterol, which is necessary for neuronal integrity and synaptic plasticity (Schultz et al., 2018). Inhibition of brain cholesterol by lovastatin promotes Aβ formation and development of AD in mice (Strandberg et al., 1999). Inhibition of brain cholesterol by statins attenuates neuronal myelination, causing worsening of AD neuropathology (Alsubaie et al., 2022). In addition, prolonged use of statins reduces the neuroprotective CQ10, leading to mitochondrial dysfunction, oxidative stress, and enhancement in Aβ formation (Alsubaie et al., 2022). Therefore, prolonged use of statins is implicated in the pathogenesis of AD by different mechanisms, as shown in Figure 3.

**Figure 4:**
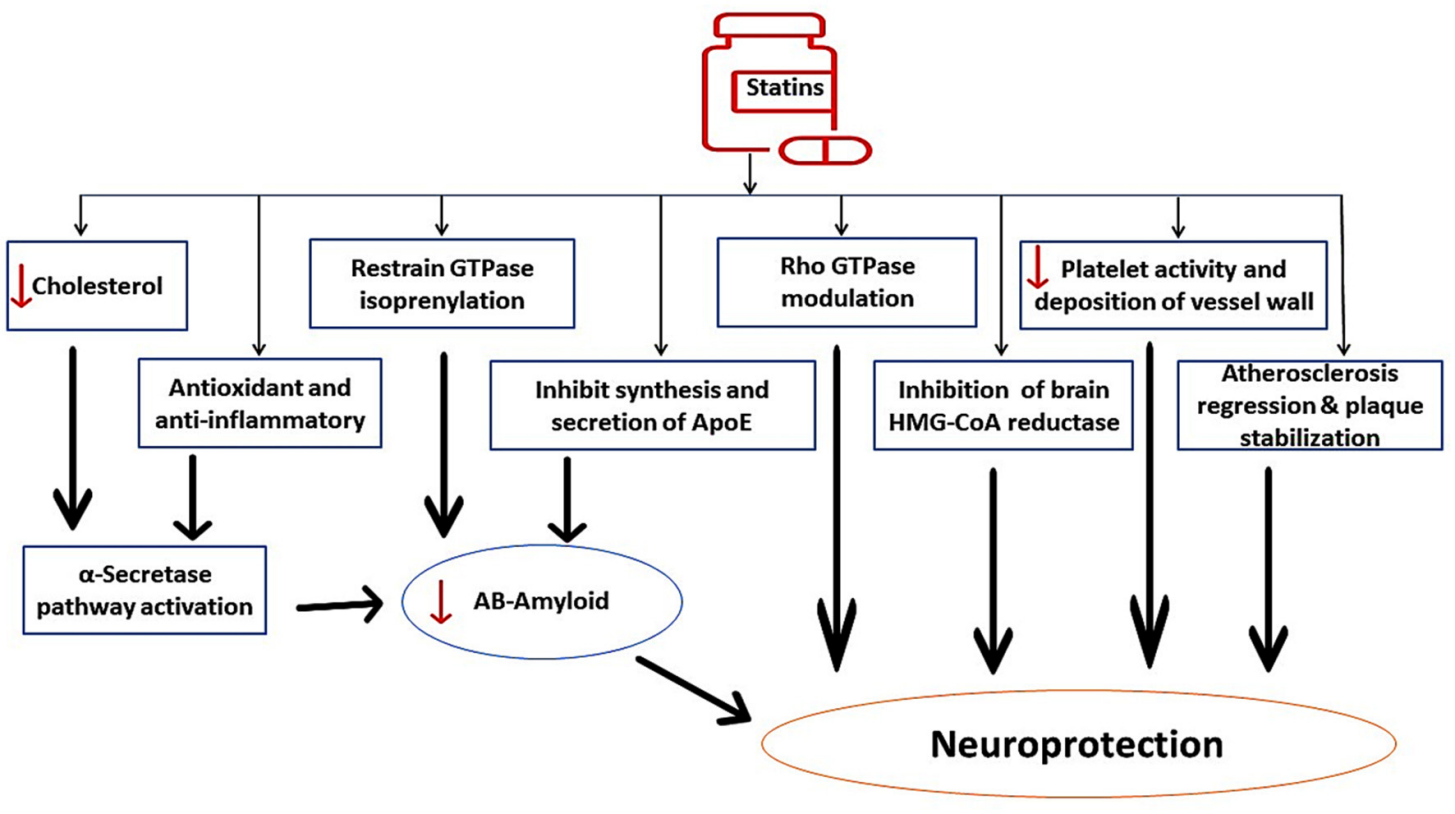
The beneficial role of statins against AD neuropathology. Statins improve α-secretase activity, which enhances the production of neuroprotective soluble Aβ with subsequent reduction in neurotoxic Aβ. Statins inhibit brain HMG-CoA reductase, reduce platelet activity in the cerebral vasculature, stabilize atherosclerotic plaques, and modulate Rho GPTase activity. Statins have indirect neuroprotective effects against AD neuropathology by targeting Aβ.

In contrast, long-term use of statins may have beneficial effects on AD neuropathology through modulation of ApoE. Thus, early treatment with statins could be therapeutically effective against AD neuropathology (Prince et al., 2014). Evidence from clinical studies confirmed the protective role of statins against the development and progression of AD (Haag et al., 2009; Lin et al., 2015). The fundamental mechanism for the protective effects of statins against AD development is linked to the inhibition of Aβ formation by suppressing ApoE4 (Bagheri et al., 2020). In addition, statins improve α-secretase activity, which enhances the production of neuroprotective soluble Aβ with subsequent reduction in neurotoxic Aβ. Statins have direct neuroprotective effects through inhibition of brain HMG-CoA reductase, reduction of platelet activity in the cerebral vasculature, stabilization of atherosclerotic plaques, and modulation of Rho GPTase activity. In addition, statins have indirect neuroprotective effects against AD neuropathology by targeting Aβ (Haag et al., 2009; Lin et al., 2015; Bagheri et al., 2020), as shown in Figure 4. These findings indicated a potential controversy regarding statin use and AD risk.

**Figure 5:**
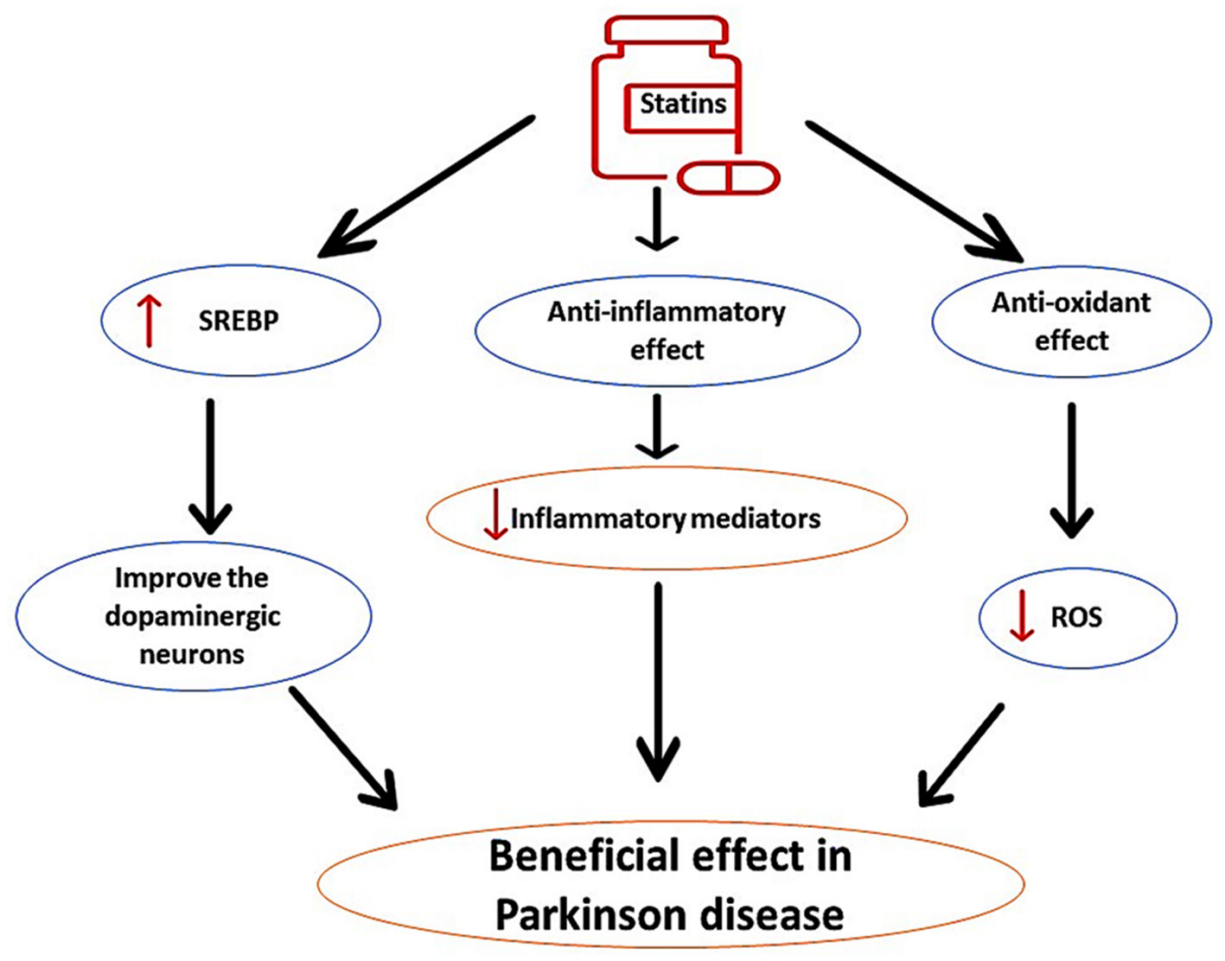
The protective role of statins in PD. Statins, through their anti-inflammatory and antioxidant effects, as well as by increasing sterol regulatory element-binding protein (SREBP), improve the activity of dopaminergic neurons, can reduce the pathogenesis of PD.

PD is the second-most common progressive neurodegenerative disease caused by degeneration of dopaminergic neurons in the substantia nigra (Al-Kuraishy et al., 2023c). PD is more frequent in subjects aged >65 years with specific classical features including bradykinesia, resting tremor, rigidity, and postural instability (Alrouji et al., 2024). It has been reported that long-term use of statins for prevention of primary and secondary outcomes in patients with cardiovascular diseases may affect the pathogenesis of PD in a bidirectional way and could be advantageous or disadvantageous (Al-Kuraishy et al., 2023a). These findings raise a conflicting role for statins in PD neuropathology. Different studies have highlighted that statins have protective effects against PD incidence, independent of cholesterol-lowering effects (Roy and Pahan, 2011). Findings from preclinical studies revealed that lipophilic simvastatin attenuates PD development in mice (Tang et al., 2019). Clinically, prolonged use of statins reduces PD risk (Wahner et al., 2008). It has been shown that statins through their anti-inflammatory and antioxidant effects as well as by increasing sterol regulatory element-binding protein (SREBP), improve the activity of dopaminergic neurons (Wahner et al., 2008), as shown in Figure 5.

In contrast, lipophilic statins may increase PD risk within 2.5 years by reducing the neuroprotective effect of cholesterol (Liu et al., 2017). Interestingly, short-term use of statins for < 1 year augments PD risk in a dose-dependent manner by inhibiting dopamine transporters, though long-term use of statins did not have this effect (Jeong et al., 2019). Therefore, the effects of statins on and PD risk and neuropathology depend on the stage of PD and the duration of statin therapy. In a similar manner, statins affect other neurodegenerative diseases.

Taken together, the effects of statins on the most common neurodegenerative diseases, including AD and PD, appear conflicting. The underlying causes of the conflicting effects of statins on AD and PD need to be addressed by prospective studies.

## 5 Role of statins in ALS

### 5.1 Protective effects

Many studies have highlighted the protective effects of statins against ALS risk (Weisskopf et al., 2022; Kreple et al., 2023). Lovastatin 40 mg/kg attenuates ALS risk in a mouse model by 28% (Kreple et al., 2023). Lovastatin has an inverse relationship in a dose-dependent manner with the risk of motor neuron disease and delays the onset of ALS in transgenic mice (Kreple et al., 2023). A longitudinal case–control study observed that prolonged use of statins >3 years reduces ALS risk (Weisskopf et al., 2022), suggesting that long-term use of statins had a protective role against the development and progression of ALS.

Preclinical findings showed that simvastatin can reduce the activation of astrocytes and microglia and inhibit the release of pro-inflammatory cytokines in C6 glioma cells subjected to the effects of LPS (Zheng et al., 2018). Simvastatin regulates the microglia immune response to ALS (Zheng et al., 2018). Inhibition of microglia and development of neuroinflammation by statins leads to a neuroprotective effect by attenuating the development of different neurodegenerative diseases including ALS (Bagheri et al., 2020). Interestingly, administration of atorvastatin 10 mg/kg/day for 4 weeks attenuates the degeneration of motor neurons in mice (Iwamoto et al., 2009). Atorvastatin inhibits denervation atrophy and motor neuron loss by 30% (Iwamoto et al., 2009).

In a clinical setting, a case–control study on 948 ALS patients and matched 1,000 healthy controls revealed that statin use for 3 years before the onset of ALS symptoms was protective against ALS neuropathology (Weisskopf et al., 2022). Numerous studies have indicated the neuroprotective effects of statins against various types of neurodegenerative diseases, including ALS, by inhibiting neurotoxicity (Bösel et al., 2005; Wolozin et al., 2007; Bagheri et al., 2020). A retrospective study on 650 ALS patients and 365 matched healthy controls revealed that 65% of ALS patients have dyslipidemia, mainly hypercholesterolemia, that is correlated with disease severity (Chełstowska et al., 2021). Therefore, hypercholesterolemia followed by mixed dyslipidemia is an important risk factor in the general population for increased ALS risk, and treating these metabolic disorders with statins may reduce the incidence of ALS. Dyslipidemia with increasing cholesterol and LDL levels augments ALS risk (Mariosa et al., 2017).

A follow-up of dyslipidemic patients for >20 years showed that elevation of one unit of LDL was linked with increased ALS risk (Mariosa et al., 2017). Therefore, alterations in the lipid profile are regarded as biomarkers before the onset of ALS. In addition, hypercholesterolemia induces the development and progression of ALS neuropathology (Chen et al., 2018; Bandres-Ciga et al., 2019; Zeng and Zhou, 2019). Thus, total cholesterol and LDL are potential risk factors involved in the pathogenesis of ALS. A large prospective study on 502,409 ALS patients showed that a high cholesterol/HDL ratio and high LDL and ApoA serum levels were associated with increased ALS risk (Thompson et al., 2022). Herein, the premorbid metabolic status may be involved in the pathogenesis of ALS, and the use of statins may improve the metabolic profile and reduce ALS risk. Furthermore, lipid homeostasis is highly dysregulated in the spinal cord gray matter, mainly in the motor neurons, leading to progressive neuronal injury. An increase in cholesterol ester is linked with the degeneration of motor neurons in ALS patients (Dodge et al., 2020).

A similar finding in the ALS mouse model revealed a significant downregulation of cholesterol biosynthesis in the motor neurons (Dodge et al., 2020). Therefore, dysregulation of lipid metabolism in the spinal cord contributes to the pathogenesis of ALS. Hence, restoration of lipid homeostasis by statins could prevent ALS development. Moreover, the protective effects of statins against ALS risk may be sex-dependent (Nefussy et al., 2011). A retrospective study illustrated that the use of statins reduced ALS risk in women, only signifying the sex-dependent effects of statins (Nefussy et al., 2011). The protective role of statins against ALS is listed in Table 2.

**Table 2:** The protective effects of statins against ALS.

**Type of the study**	**Findings**	**References**
Preclinical	Lovastatin 40 mg/kg attenuates ALS risk in mouse models by 28%	Kreple et al., 2023
Preclinical	Simvastatin reduces the activation of astrocytes and microglia and inhibits the release of pro-inflammatory cytokines in C6 glioma cells	Zheng et al., 2018
Preclinical	Administration of atorvastatin 10 mg/kg/day for 4 weeks attenuates the degeneration of motor neurons in mice	Iwamoto et al., 2009
Longitudinal study	Prolonged use of statins >3 years reduces ALS risk	Weisskopf et al., 2022
Clinical studies	Statins have protective effects against neurodegenerative diseases including ALS by inhibiting neurotoxicity	Bösel et al., 2005; Wolozin et al., 2007; Bagheri et al., 2020

**Figure 6:**
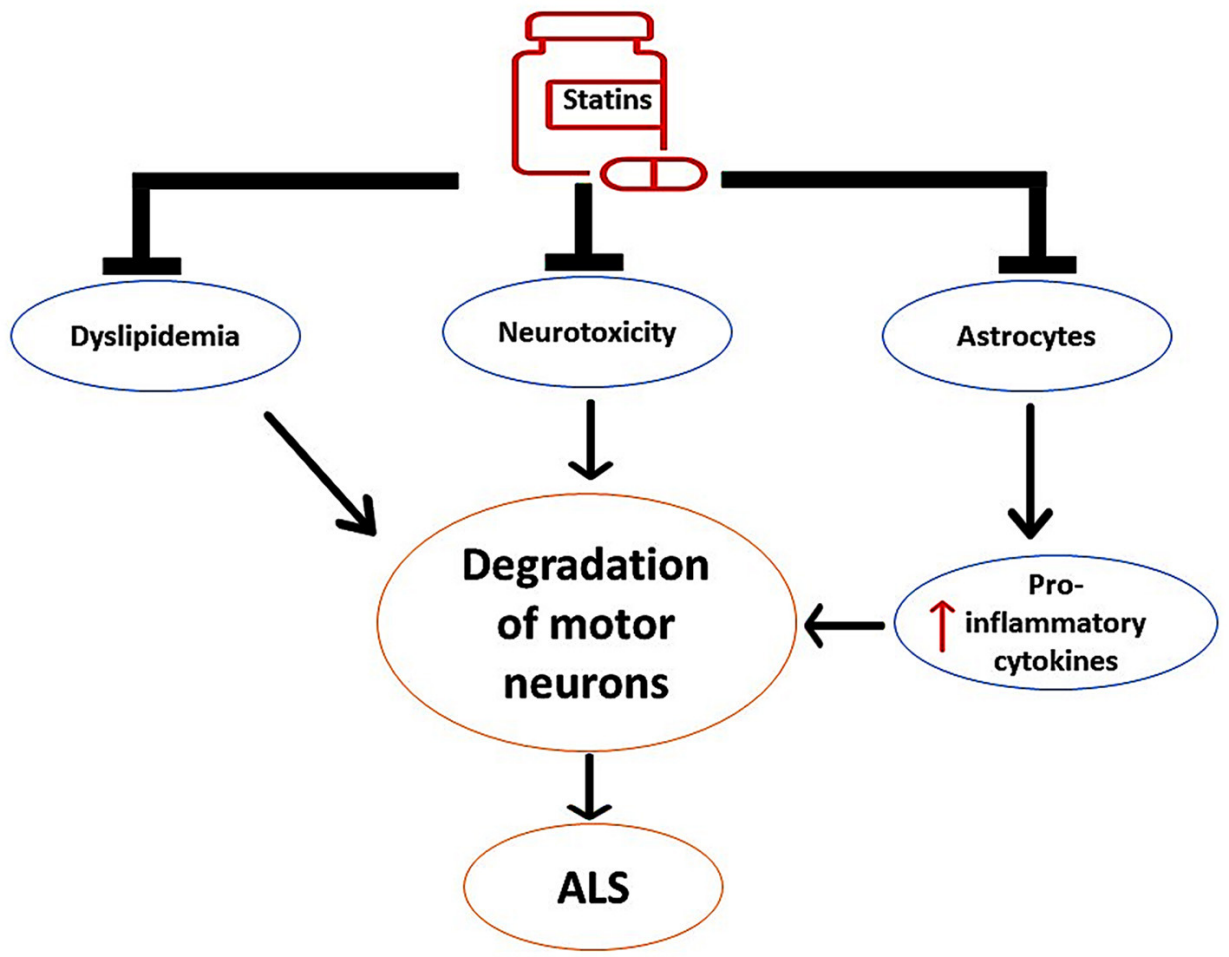
The neuroprotective effects of statins against the development of ALS. Statins inhibit the activity of overactivated astrocytes and the release of pro-inflammatory cytokines. Statins attenuate the progression of neurotoxicity, thereby preventing the degeneration of motor neurons. In addition, statins attenuate the development of dyslipidemia, which is implicated in the degeneration of motor neurons.

These findings proposed the neuroprotective effects of statins against the development and progression of ALS by regulating dyslipidemia and inflammatory changes, as shown in Figure 6.

### 5.2 Hazardous effects

It has been observed that statins may adversely affect the incidence and progression of ALS (Gaimari et al., 2022). A population-based study revealed that long-term use of statins is associated with the acceleration of ALS severity (Mariosa et al., 2020). Evidence from previous studies indicated that prolonged use of statins may be associated with the development of ALS-like syndrome (Edwards et al., 2007; Colman et al., 2008). Statins promote the progression of peripheral neuropathy and rhabdomyolysis, though the association of statins with upper motor lesions was disproportionate (Edwards et al., 2007). Data obtained from a retrospective study proposed that statin use increases ALS risk compared to placebo (Colman et al., 2008). A population-based case–control study on 2,475 ALS patients and 12,375 healthy controls illustrated that statins increase ALS risk in women (Mariosa et al., 2020). Small muscle mass and low-statin metabolizing enzymes in women may explain the sex difference in the effect of statins (Group, 2008). The neurotoxic effects of statins may increase ALS risk in women mainly in the first year from the onset of ALS (Skajaa et al., 2021). A recent population-based cohort study revealed that genetic predisposition for statin therapy is linked with augmentation of ALS risk (Wang et al., 2023). Thus, statins may increase ALS, independent of cholesterol-lowering effects, which gives an insight into a new avenue regarding the role of statins in ALS.

It has been illustrated that not all statins produce detrimental effects on motor neurons, though a class-dependent effect was postulated. A previous *in vitro* study demonstrated that 7-day exposure of motor neurons to fluvastatin has less effects on spinal motor neurons in a dose-dependent manner (Murinson et al., 2012). In comparison, much higher concentrations of other statins are required to produce neurotoxic effects. However, this neurotoxic effect was not observed on cortical neurons or Schwann cells (Murinson et al., 2012). However, hydrophilic pravastatin, which has a pharmacokinetic profile distinct from that of lipophilic fluvastatin, produces less toxic effects on the spinal motor neurons (Murinson et al., 2012). Therefore, class-dependent effects of statins by specific mechanisms may be implicated in the pathogenesis of ALS. Lipophilic statins have greater effects on the initiation of ALS neuropathology compared to hydrophilic statins (Golomb et al., 2018). However, statins affect ALS through cholesterol-dependent and cholesterol-independent mechanisms. Inhibition of neuronal cholesterol by statins interferes with the lipid raft, which mediates the neuronal pro-survival pathway (Fracassi et al., 2019).

The cholesterol-independent mechanism of statins is through the reduction of mevalonate, which is essential for neuronal survival (Moutinho et al., 2017). Therefore, statins may attenuate the survival of motor neurons through the suppression of pro-survival signaling. In addition, statins induce the expression of pro-apoptotic pathways such as Bcl-2 and FOXO3a, leading to neuronal apoptosis (Wood et al., 2013). Of note, statins affect inflammatory signaling that distorts G-protein and carnitine palmitoyl transferase in the mitochondrial membrane, leading to oxidative stress (Yanae et al., 2011). In addition, statins decrease the binding of Rho and Ras proteins to the cell membrane, which is intricately involved in neuronal endocytosis. In addition, statins induce apoptosis in glioma cell lines through Akt/ERKI/2 signaling (Yanae et al., 2011). In addition, statins that interfere with musculoskeletal metabolism may increase ALS risk (Gaimari et al., 2022). Therefore, ALS could be a potential adverse effect of statin therapy.

Nakamura et al. (2022) observed that high HDL in ALS patients predicts poor prognosis for all patients, though low LDL augments ALS risk in women only. A retrospective study on 78 ALS patients showed that high HDL levels and low LDL levels are correlated with hypermetabolism in ALS patients (Nakamura et al., 2022). High HDL activates the expression of mechanistic target of rapamycin (mTOR) via phosphatidylinositol 3 kinase (PI3K), causing inhibition of autophagy and augmentation of ALS neuropathology (Wang and Peng, 2012). Inhibition of autophagy accelerates the accumulation of TDP-43, increasing the pathogenesis of ALS (Tang et al., 2019). In ALS, muscle metabolism is shifted toward the lipid rather than glycolytic pathway. However, high HDL induces muscle glycolysis and reduces β-oxidation in muscles, thereby producing a protective effect (Ferri et al., 2017). In this state, the lipid-lowering effect of statins by increasing HDL and reducing LDL may be implicated in the pathogenesis of ALS.

The mechanism for induction of ALS neuropathology by statins may be related to the dysregulation of liver X receptor signaling (LXR) signaling in the motor neurons (Beltowski, 2010). Statins suppress the synthesis of endogenous LXR agonist oxysterols and reduce the expression of the LXR gene. Of note, mice lacking the *LXR* gene experience an ALS phenotype. Statin-induced inhibition of *LXR* gene expression promotes circulating sterols, which are neurotoxics (Beltowski, 2010). Therefore, LXR agonists may attenuate statin-induced ALS. In this state, statin therapy might not be safe in ALS patients (Zinman et al., 2008), although an observational study indicated that statin therapy is rarely associated with ALS risk (Golomb et al., 2009).

Moreover, brain cholesterol and even dyslipidemia are considered protective factors against ALS neuropathology (Dupuis et al., 2008; Yoshii et al., 2010). A case–control study on 369 ALS patients and 286 healthy controls observed that dyslipidemia and a high LDL/HDL ratio prolong the survival of ALS patients by 12 months (Dupuis et al., 2008). It has been shown that hypermetabolism is augmented in ALS patients; thus, reduction of cholesterol by statin therapy may reduce the nutritional status of muscles and neurons, leading to the progression of the pathogenesis of ALS (Dupuis et al., 2008). Yoshii et al. proposed that high energy demand in ALS can reduce the LDL/HDL ratio, which correlated with respiratory complications in ALS patients (Yoshii et al., 2010). The hazardous effects of statins are listed in Table 3.

**Table 3:** The hazardous effects of statins on ALS.

**Type of the study**	**Findings**	**References**
Clinical	Long-term use of statins is associated with the acceleration of the severity of ALS	Mariosa et al., 2020
Clinical	Prolonged use of statins may be associated with the development of ALS-like syndrome	Edwards et al., 2007
A retrospective study	Statin use increases ALS risk compared to placebo	Colman et al., 2008
A cohort study	Statin use in women may increase ALS risk mainly in the first year from onset of ALS	Skajaa et al., 2021
Preclinical	Fluvastatin reduces effects on spinal motor neurons in a dose-dependent effect	Murinson et al., 2012
Preclinical	Lipophilic statins have greater effects on the initiation of ALS neuropathology compared to hydrophilic statins	Golomb et al., 2018
A retrospective study	High HDL level and low LDL level due to prolonged use of statins are correlated with hypermetabolism in ALS patients	Nakamura et al., 2022
Preclinical	Induction of ALS neuropathology by statins may be related to the dysregulation of LXR signaling in the motor neurons	Beltowski, 2010

**Figure 7:**
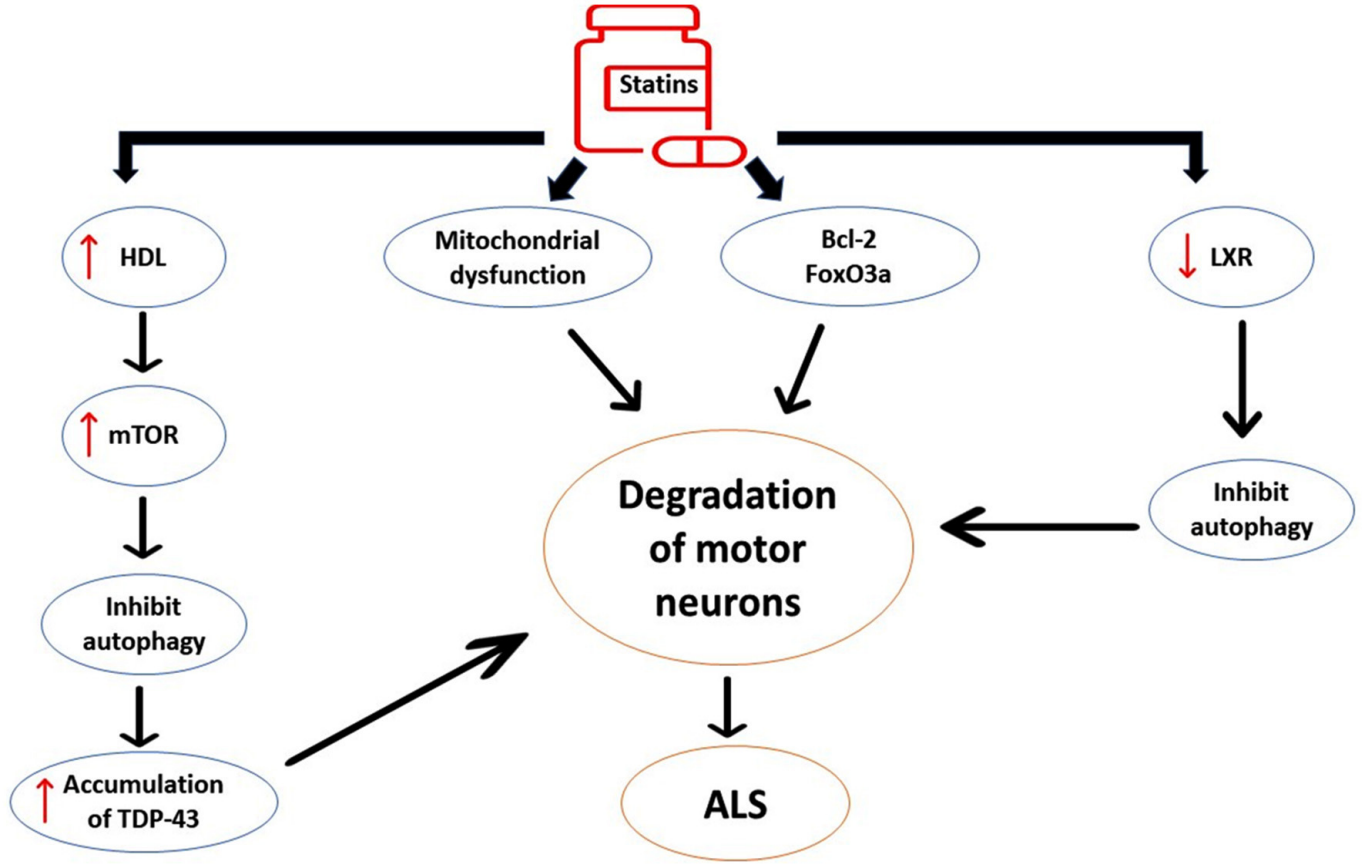
The detrimental effects of statins on ALS neuropathology. Statins induce the expression of pro-apoptotic pathways such as Bcl-2 and FOXO3a, leading to neuronal apoptosis. Statins affect inflammatory signaling that distorts G-protein and carnitine palmitoyl transferase in the mitochondrial membrane, leading to oxidative stress and mitochondrial dysfunction. Statin-induced high HDL activates the expression of the mechanistic target of rapamycin (mTOR), causing inhibition of autophagy and augmentation of ALS neuropathology. Statins inhibit the expression of liver X receptor signaling (LXR) signaling in the motor neurons. These changes induced by statin use trigger the degeneration of motor neurons and development of ALS.

Taken together, statins may accelerate ALS neuropathology by alteration of lipid profile and dysregulation of neuronal inflammatory signaling (Figure 7).

### 5.3 Neutral effects

It has been perceived that metabolic changes such as diabetes and dyslipidemia are common in ALS patients due to a derangement of energy balance and hypermetabolism (Schumacher et al., 2020). A prospective cohort study showed that statin use did not affect the overall survival of ALS patients (Schumacher et al., 2020); thus, statin therapy for the management of dyslipidemia should not be discontinued in ALS patients. A retrospective study included 459 ALS patients, 72 of them were on treatment with statins at the onset of the disease, and there was no significant association between statins and increased ALS risk (Drory et al., 2008). A pooled analysis of observational studies for the association between statin use and ALS risk was inconclusive (Macías Saint-Gerons and Castro, 2019). A systematic meta-analysis found no association between statin use and ALS risk (Chang et al., 2021). A recent meta-analysis and systematic review of 13,890 ALS patients from eight clinical studies revealed no significant association between prolonged use of statins and ALS risk (Nabizadeh et al., 2022). Other systematic reviews and meta-analyses illustrated that prolonged use of statins was not linked with ALS risk (Hu and Ji, 2022). Therefore, most recent studies and meta-analysis reviews indicated that statin use was not associated with disease progression or amelioration of survival in ALS patients.

## 6 Discussion

Statins as a risk factor for the development of ALS should be explained with caution, as the increasing use of statins has dramatically increased from 5% in 1991 to 40% in 1998 (Toft Sørensen and Lash, 2009). Many adverse effects related to statin medications are reported from uncontrolled clinical trials and non-randomized studies (Cameron et al., 2015). The evidence for statin-induced neurotoxic effects is highly limited (Samant and Gupta, 2021). ALS patients are commonly associated with dyslipidemia, liver steatosis, and other metabolic disorders (Colman et al., 2008; D'Amico et al., 2021). However, it is difficult to verify the causal relationship between ALS and metabolic disorders as most of the reported studies were observational. In 2007, the WHO declared that prolonged use of statins may induce the development of ALS-like syndrome and may increase ALS risk (Edwards et al., 2007), and this was the first report to suggest the potential link between statin use and ALS risk. The WHO database regarding this association is called Vigibase, which contains 4,000,000 subjects who reported the adverse effects of different medications, including statins (Macías Saint-Gerons and Castro, 2019).

Initially, a cohort study involving 172 ALS patients revealed that 25% of ALS neuropathologies is related to long-term use of statins (Edwards et al., 2007). Colman et al. (2008) in 2008 described an identical association between statin use and ALS risk, depending on the Vigibase database. Despite of this evidence, there is no valid and robust clinical evidence explaining the causal relationship between statin therapy and ALS risk. In fact, the surveillance databases give clues and provide an idea regarding the connection between statin therapy and ALS that should be confirmed by preclinical and clinical studies regarding the re-analysis of databases. In addition, the analysis of databases should be in relation to the incidence and prevalence of ALS in certain regions worldwide regarding the prevalence of statin use. For example, a pooled analysis of clinical trials showed an association between statin use and risk for development of ALS. However, these findings did not provide robust and strong clinical evidence for this association due to improper description of study criteria (Rothman et al., 2008). Moreover, misdiagnosis and misclassification of ALS cases included in clinical trials and observational studies affect the outcomes and validity of different studies (Sørensen et al., 2006). The biased null-hypothesis in some studies could be an important cause for the negative association between statin use and ALS risk (Sørensen et al., 2006). Therefore, systematic reviews and meta-analyses illustrated no association between long-term use of statins and ALS risk (Chang et al., 2021; Hu and Ji, 2022; Nabizadeh et al., 2022). Currently, small incidence of ALS despite the high prevalence of statins use supports a null-hypothesis between long-term use of statins and ALS risk (Colman et al., 2008).

Interestingly, measures of ALS progression should be evaluated in patients with prolonged use of statins. Of note, magnetic resonance tomography (MRI) of the CNS and CSF biomarkers can predict the severity of ALS progression (Spinelli et al., 2020; Dreger et al., 2022). Functional staging of ALS is commonly measured by King's clinical staging and Milano–Torino (MiToS). The MiToS system uses six stages, from 0 to 5, and is based on functional ability, as assessed by the ALS Functional Rating Scale-Revised (ALSFRS-R), with stage 0 being normal function and stage 5 being death. The King's system uses five stages, from 1 to 5, and is based on disease burden, as measured by clinical involvement and significant feeding or respiratory failure, with stage 1 being symptom onset and stage 5 being death (Fang et al., 2017). King's clinical staging has higher resolution in the early stage of ALS; however, MiToS staging has a great resolution for the late stage of ALS (Fang et al., 2017). Therefore, both staging systems are recommended to evaluate the clinical staging of ALS patients.

In addition, the ALS-functional scale can measure clinical motor deficits in ALS patients (Spinelli et al., 2020; Dreger et al., 2022). Despite these measures, there is little agreement as to what measures to use in a given clinical setting in ALS patients since ALS is regarded as a protean disease that may progress rapidly or slowly. In addition, the prognosis of ALS is poor, which should be discussed with patients regarding survival and response to the current treatments (van Eenennaam et al., 2020). The personalized ENCALS survival prediction model dependably estimates the personalized prognosis of ALS patients without destroying hope and anxiety. The communication guide supports physicians in discussing the personalized prognosis for ALS patients and their families (van Eenennaam et al., 2020).

Taken together, these limitations in the explanation of data result in controversial outcomes with opposing mechanisms regarding the association between statin use and ALS neuropathology. The present review had many limitations, such as the limitation of prospective studies, and most of the published studies were subjected to many limiting factors, such as bias and heterogeneity. Therefore, reaching a final conclusion regarding the exact role of statins in ALS remains difficult. Therefore, large-scale prospective studies with proper ALS diagnostic criteria to elucidate the connotation between statins use and ALS are recommended in this regard.

## 7 Conclusions

ALS is a neurodegenerative disease characterized by muscle weakness, muscle twitching, and muscle wasting due to progressive neurodegeneration of motor neurons located in the brain, motor cortex, and spinal cord. It has been shown that prolonged use of statins may induce the development of ALS-like syndrome and may increase ALS risk. Conversely, results from preclinical and clinical studies highlighted the protective role of statins against ALS neuropathology. Recently, meta-analyses and systematic Reviews showed no association between long-term use of statins and ALS risk. Therefore, there is a strong controversy regarding this association. Statins as a risk factor for the development of ALS should be explained with caution, as the use of statins has significantly increased. At this time, the small incidence of ALS despite the high prevalence of statins use supports a null-hypothesis between long-term statin use and ALS risk. Collectively, these precincts in the explanation of data result in controversial outcomes with contrasting mechanisms regarding the association between statin use and ALS neuropathology. Consequently, large-scale prospective studies involving patients with appropriate ALS diagnostic criteria to explicate the association between statin use and ALS are recommended in this regard.

## Author contributions

HA-k: Supervision, Writing – original draft. MJ: Supervision, Writing – original draft. GS: Project administration, Writing – review & editing. HM: Supervision, Writing – review & editing. AA-G: Writing – original draft, Writing – review & editing. AA: Writing – review & editing. SJ: Writing – original draft, Writing – review & editing. AS: Software, Writing – review & editing. MA: Funding acquisition, Software, Writing – review & editing.
